# Molecular Detection of SFGR in Ticks Collected from Yaks in Jiulong County of Sichuan Province, China

**DOI:** 10.3390/ani15070975

**Published:** 2025-03-28

**Authors:** Lili Hao, Liangquan Zhu, Chendong Xiao, Rihong Jike, Kelei Zhou, Yinglin Li, Quwu Jise, Chang Wang, Lu Deng

**Affiliations:** 1 College of Animal Husbandry and Veterinary Medicine, Southwest Minzu University, Chengdu 610041, China; leelee_hao@126.com; 2 China Institute of Veterinary Drug Control (IVDC), Beijing 100081, China; zhuliangquan_ivdc@126.com; 3 Center for Animal Disease Control and Prevention in Xiangcheng County, Xiangcheng County Bureau of Agriculture, Xuchang 452670, China; xcd1078501821@163.com; 4 Agricultural and Rural Bureau of Liangshan Yi Autonomous Prefecture of Sichuan Province, Liangshan 615000, China; 18788911610@163.com (R.J.); 13881571002@163.com (K.Z.); 13981526204@163.com (Y.L.); 18882856822@163.com (Q.J.); 5 College of Computer Science, Chengdu Normal University, Chengdu 611130, China; 6 College of Culinary and Food Science Engineering, Sichuan Tourism University, Chengdu 610100, China

**Keywords:** spotted fever group rickettsiae, *Bos grunniens*, tick, high prevalence, Jiulong County

## Abstract

This study investigated tick species and spotted fever group rickettsiae (SFGR) infection rates in ticks from yaks in Jiulong County, Sichuan Province, China. Firstly, ticks collected from yaks were meticulously classified through morphological identification. Subsequently, the total DNA of ticks was extracted, and specific partial sequences of their *ITS-2* region, as well as the *ompA* and *ompB* genes of SFGR, were amplified using PCR. The positive PCR products were sequenced and compared thoroughly against the NCBI database. A phylogenetic analysis was conducted for *ITS-2*, *ompA*, and *ompB* to determine the species of tick and the SFGR. The results revealed that a total of 585 ticks were collected, with the most abundant species being *R. microplus*, which accounted for 52.65% (308/585) of the total and was followed by *I. ovatus* (32.99%, 193/585), *I. acutitarsus* (8.89%, 52/585), and *D. everestianus* (5.50%, 32/585). Furthermore, SFGR was detected in 63.93% (374/585) of the ticks. Notably, the infection rate of SFGR in semi-agricultural and pastoral areas was significantly higher at 70.60%, compared to 45.10% in pastoral areas (*p* < 0.01). Overall, this study marks the first investigation into the prevalence of SFGR in Jiulong County and highlights the high infection rate of SFGR in ticks, with *R. raoultii* being the predominant species.

## Simple Summary

This study presents the first epidemiological survey of tick-borne spotted fever pathogens (SFGR) in yaks from Jiulong County, Southwest China. By analyzing 585 ticks collected across pastoral and semi-agricultural zones through morphological and molecular methods, we identified four tick species, predominantly *Rhipicephalus microplus* (52.65%). Strikingly, 63.93% of ticks carried SFGR pathogens, with *Rickettsia raoultii* as the dominant species. Infection rates were significantly higher in semi-agricultural areas (70.60%) than in pastoral zones (45.10%), revealing elevated disease risks in human-modified landscapes. These findings provide critical insights into SFGR ecology at human–animal interfaces and underscore the urgency of surveillance in transitional ecosystems.

## 1. Introduction

Ticks are significant obligate blood-feeding ectoparasites that attach to the body surface of various hosts, including mammals, birds, and reptiles. They function critically as natural reservoirs and transmission vectors for a wide array of pathogens, including bacteria, viruses, and protozoa, many of which can infect humans and animals, leading to numerous zoonotic diseases. In recent years, with global warming and the continuous expansion of human activities, the incidence of tick-borne diseases has been increasing. Therefore, understanding the distribution of tick populations and their pathogen carriage is vital for the prevention and control of tick-borne diseases, which helps ensure the health of animals and humans and improves the overall level of public health safety [1].

Spotted fever group rickettsiae (SFGR) are obligate intracellular bacteria primarily transmitted through tick bites, causing a range of diseases in humans such as Rocky Mountain spotted fever and Mediterranean spotted fever [2]. The disease typically presents with sudden fever, accompanied by headache and malaise, and skin manifestations such as rashes and eschars. Clinical symptoms can vary widely. While most patients experience mild symptoms, a minority may develop severe symptoms that can be life-threatening [3,4,5,6]. For example, *Rickettsia raoultii* was first detected in 1999 in *Haemaphysalis concinna* and *Hyalomma asiaticum* collected from the former Soviet Union border region [7]. In 2008, *R. raoultii* was identified as a new *Rickettsia* based on genomic and serological characteristics [8]. In 2012, *R. raoultii* was first detected in *Dermacentor silvarum* in Xinjiang, China. *R. raoultii* has a wide geographic distribution, with reports of its detection in different tick species in Germany, France, and other European countries, as well as in South Korea, Mongolia, and other Asian countries [9,10,11,12]. In China, there have been several reports of human infections with *R. raoultii* following tick bites. For instance, in 2012, *R. raoultii* was detected in two patients in Mudanjiang, China. The *Rickettsiae* isolated from these two patients were homologous to those found in *D. silvarum* ticks collected from the patients’ surrounding environments [13]. From 2015 to 2016, Chinese scholars detected *R. raoultii* in patient samples from Shandong, Henan, and Inner Mongolia (26/1295), successfully isolating *R. raoultii* from these samples [14].

Jiulong County, located in the western part of Sichuan Province and southeast of the Ganzi Tibetan Autonomous Prefecture, covers an area of 6770 square kilometers and has a continental plateau climate. The county is characterized by its extensive grasslands (approximately 334,400 hectares), which account for 49.40% of the total area, making it a typical semi-agricultural and semi-pastoral region. Yaks (*Bos grunniens*) are the primary economic animals in Jiulong County, with a population of approximate 59,436 as of December 2020. Currently, there are no reports on the ticks or the prevalence of SFGR in this area. Therefore, this study aims to investigate the tick species infesting yaks in Jiulong County and assess their infection status with SFG*R*. The findings would provide valuable scientific evidence for ensuring the health of local residents, enhancing public health levels, and supporting the sustainable development of animal husbandry in this region.

## 2. Materials and Methods

### 2.1. Ethics Statement

This study was approved by the Animal Ethics Committee of Southwest Minzu University (approval NO. AECSWU2020-12).

### 2.2. Sample Collection and Ticks Morphological Classification

Ticks were collected from the body surface of yaks in Tanggu (coordinate: 30.417778, 101.283333), Sanyanlong (coordinate: 28.7869156, 101.2811314), and Xia’er (coordinate: 29.0068878, 101.5157275), townships in Jiulong County, Sichuan Province, China from May to September 2020. In each township, ticks were gathered from 2 to 5 villages. The collection focused on the ears, face, and neck of each yak, and the ticks were then placed into tubes with moist cotton. After morphological classification, all ticks were stored in 75% ethanol at 4 °C. The feeding ticks were identified based on their morphological characteristics, such as false head base, basal process, whisker limb, scutellum, margin stack, stomatal plate, foot and anal groove, using standard taxonomic keys [15] under a stereo microscope. This morphological identification was then complemented with the molecular method to accurately determine the species.

### 2.3. DNA Extraction, PCR Amplification, and Phylogenetic Analysis

All ticks were sectioned longitudinally and homogenized in ddH_2_O. The homogenates were then centrifuged for 5 min at 8000× *g*. The total DNA was extracted from all samples using the TIAN amp Genomic DNA Kit (TIANGEN Biotech Co., Ltd., Beijing, China, Cat. No. DP304) following the manufacturer’s instructions. The extracted DNA was subsequently stored at −20 °C for tick molecular identification and detection of SFGR spp. Primer sequences are listed in Table 1.

To identify the species of ticks, a partial sequence of the ribosomal *ITS-2* gene, approximately 750–1800 nucleotides (nt) in length, was amplified using primers (ITS2-F and ITS2-R), as described by Lv et al., 2014 [16]. In addition, all samples were subjected to PCR assays targeting the *ompA* gene of *Rickettsia* (347 bp), as described by Li et al., 2016 [17]. All samples that tested positive for *ompA* were further analyzed, targeting *ompB* (418 bp) [18]. PCR was performed in a total reaction volume of 25 μL, including 1 μL of template DNA or 1 μL of PCR product, 1 μL of each primer (10 μM), 12.5 μL of PCR Supermix (TransGen Biotech Co., Ltd., Beijing, China, Cat. No. AS111), and 9.5 µL of distilled wate*R*. After an initial denaturation for 3 min at 95 °C, 40 cycles of denaturation for 30 s at 94 °C, annealing for 30 s at 55 °C, and elongation for 30 s at 72 °C were performed, followed by a final extension step at 72 °C for 7 min. The sequences were analyzed and compared using the DNASTAR v.7.1.0 software. Nucleotide sequences were examined using the BLAST tool (BLAST+2.16.0), following the methods described by Sayers et al., 2021 [19], so as to compare them with sequences deposited in GenBank. Phylogenetic trees were constructed using the Neighbor-Joining method in MEGA 6 software, based on the *ITS-2*, *ompA* and *ompB* genes, respectively. The evolutionary distance was calculated using the Kimura 2-parameter method, with 1000 bootstrap replicates.

**Table 1:** Primer sequences used for tick and *Rickettsia* spp. identification.

Species	Primer Sequence (5′–3′)	Target Gene	Product (bp)	References
Tick	ITS2-F: ACATTGCGGCCTTGGGTCTT ITS2-R: TCGCCTGATCTGAGGTCGAC	*ITS-2*	750–1800	[16]
*Rickettsia* spp.	190.70-38s1: AAAACCGCTTTATTCACC 190.602-384r1: GGCAACAAGTTACCTCCT	*ompA*	347	[17]
	ompB4F:GTTTAATACGTGCTGCTAACCAA ompB4R: GGTTTGGCCCATATACCATAAG	*ompB*	418	[20]

### 2.4. Statistical Analysis

A Pearson *Chi*-square (*χ*^2^) test using SPSS 19.0 (IBM, New York, NY, USA) was conducted to determine the prevalence of *Rickettsia* spp. among different sampling locations and tick species, with significant differences (*p* < 0.05).

## 3. Results

### 3.1. Tick Species and Quantities

**Figure 1:**
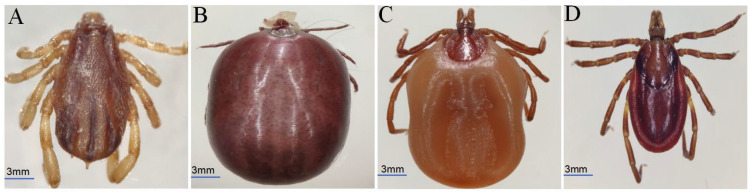
Morphological characteristics of ticks collected from yaks in Jiulong County. (**A**) Dorsal surface of *R. microplus* (male). (**B**) Dorsal surface of *I. ovatus* (female). (**C**) Dorsal surface of *I. acutitarsus* (female). (**D**) Dorsal surface of *D. everestianus* (male). Bar = 3 mm.

A total of 585 ticks were collected from three townships in Jiulong County (Xia’er, *n* = 256; Sanyanlong, *n* = 176; Tanggu, *n* = 153). Four species of ticks comprising three genera were preliminary identified (Figure 1), namely *Rhipicephalus microplus* (*R. microplus*) (*n* = 308), *Ixodes ovatus* (*I. ovatus*) (*n* = 193), *Ixodes acutitarsus* (*I. acutitarsus*) (*n* = 52), and *Dermacentor everestianus* (*D. everestianus*) (*n* = 32). *R. microplus* (52.65%) was the predominant and most prevalent species.

### 3.2. Tick Molecular Identification

After sequencing the *ITS-2* gene of the ticks, nine unique sequences were obtained through splicing and pairwise comparison. Among these, four sequences were identified as *I. ovatus* (named *I. ovatus* Jiulong 1–4), three as *I. acutitarsus* (*I. acutitarsus* Jiulong 1–3), and the remaining two as *R. microplus* (*R. microplus* Jiulong 1) and *D. everestianus* (*D. everestianus* Jiulong 1). *I. ovatus* Jiulong 1–4 clustered closely with the *Ixodes ovatus* isolate from Yunnan (KU664537), exhibiting the closest genetic relationship with a similarity of 99.07–99.16%. *I. acutitarsus* Jiulong 1–3 clustered with the *I. acutitarsus* isolate from Yunnan (KU664535), showing the closest genetic relationship and a similarity of 87.47–94.54%. *R. microplus* Jiulong 1 had the closest genetic relationship with the *R. microplus* isolates from Xiangxi (MK224585) and Jishou (MK224571) in Hunan, with a 100.00% similarity. *D. everestianus* Jiulong 1 was most closely related to the *D. everestianus* isolate from Lanzhou, Gansu (JQ737111), also with a 100.00% similarity. The sequences are available in the Supplementary Material.

### 3.3. Rickettsia spp. Detection

**Figure 2:**
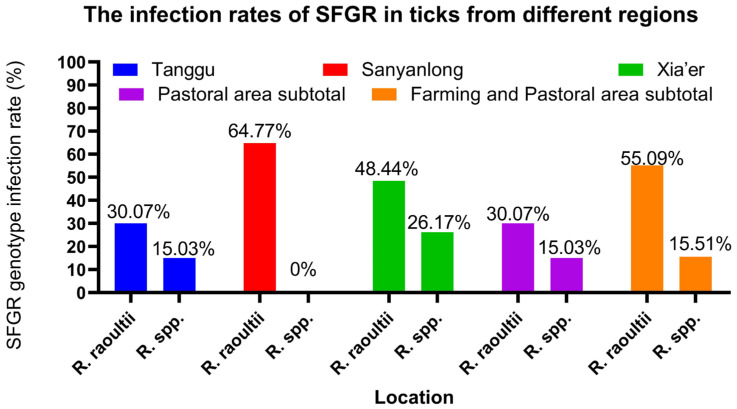
The infection rates of SFGR in ticks from different regions.

Out of the 585 tick samples, 374 tested positives for SFGR, resulting in a positive rate of 63.93%. SFGR was detected in ticks from all three towns, with strikingly high positive rates varying from 45.10% to 74.61%, as detailed in Table 2 and Figure 2. Among the three survey sites, Xia’er Town had the highest infection rate at 74.61%, followed by Sanyanlong (64.77%) and Tanggu Town (45.10%). The positive rate in pure pastoral areas was 45.10%, while in semi-agricultural and pastoral areas, it was 70.60%. The *χ*^2^ test showed that the infection rate in semi-agricultural and pastoral areas (marked with “**” in Table 2) was significantly higher than in pure pastoral areas (*p* < 0.01).

Based on the *ompA* and *ompB* genes, the 374 *Rickettsia*-positive tick samples were identified with *R. raoultii* and *Rickettsia* spp. (MZ420228). *R. raoultii* was the predominant species, accounting for 75.94% (284/374). In this study, the SFGR positive rates in *I. acutitarsus*, *I. ovatus*, *R. microplus*, and *D. everestianus* ticks were 67.31% (35/52), 63.73% (123/193), 63.64% (196/308), and 62.50% (20/32), respectively, (Table 3) with no significant difference i (*p* > 0.05).

### 3.4. Phylogenetic Analyses of Rickettsia

**Figure 3:**
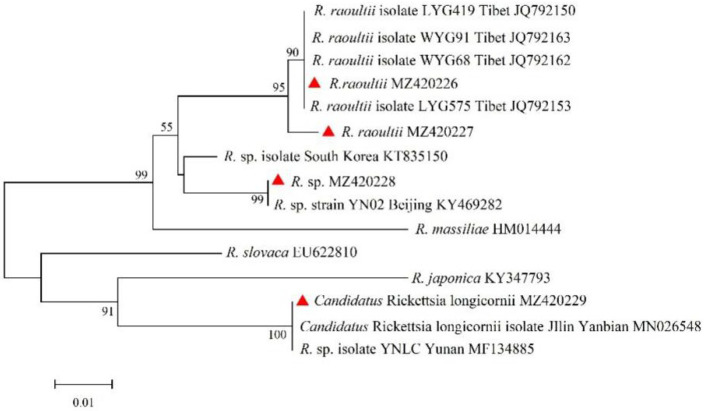
Phylogenetic tree of SFGR based on the *ompA* gene.

**Figure 4:**
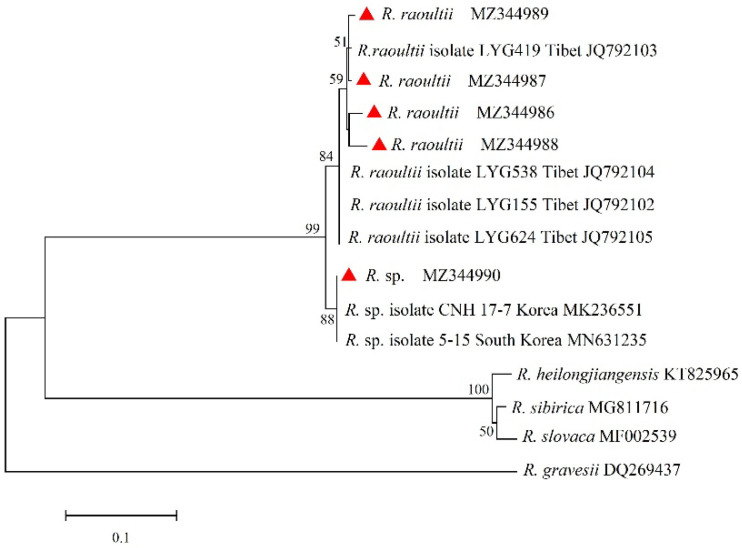
Phylogenetic tree of SFGR based on the *ompB* gene.

The *ompA* and *ompB* genes of *Rickettsia* were sequenced and compared. A total of four unique sequences of *ompA* and five unique sequences of *ompB* were obtained and deposited in GenBank with accession numbers as follows: *ompA*, MZ420226 to MZ420229; *ompB*, MZ344986 to MZ344990. For the *ompA* gene, sequences (MZ420226 and MZ420227) were clustered with *R. raoultii* isolates WYG 68 (JQ792162), WYG 91 (JQ792163), LYG 419 (JQ792150), and LYG 575 (JQ792153) from Tibet, showing sequence identities of 100% and 99.20%, respectively. The sequence (MZ420228) showed 99.59% identity to *Rickettsia* sp. isolated from Beijing (KY469282). Sequence MZ420229 was clustered with *Rickettsia* sp. isolated from Yunnan (MF134885) and *Candidatus Rickettsia longicornii* from Yanbian in Jilin (MN026548), with a sequence identity of 100%. For the *ompB* gene, sequence MZ344990 was clustered with *Rickettsia* spp. isolated CNH 17-7 (MK236551) and 5-15 (MN631235) from Korea, forming a clade with the closest phylogenetic relationship, but the highest similarity of 99.47% with *Rickettsia* sp. CNH 17-7 (MK236551) from Korea. Sequences MZ344986 to MZ344989 were clustered with *R. raoultii* strains LYG 419 (JQ792103), LYG 538 (JQ792104), LYG 155 (JQ792102), and LYG 624 (JQ792105) from Tibet, sharing 96.97–99.39% nucleotide identity with the *R. raoultii* LYG 419 (JQ792103) strain from Tibet (Figure 3 and Figure 4).

## 4. Discussion

Four tick species were identified in this study through morphological and molecular methods: *I. ovatus*, *I. acutitarsus*, *R. microplus*, and *D. everestianus* [21]. *R. microplus* was the most abundant species, accounting for 52.65% (308/585) of the total ticks collected, making it the dominant tick species in Jiulong County. *D. everestianus* and *I. acutitarsus* were only identified in yaks from Tanggu and Xia’er towns, accounting for 5.47% (32/585) and 8.89% (52/585) of the total tick population, respectively. These findings are likely related to the specific living characteristics and survival environments of each tick species, which are closely tied to local altitude and ecological conditions. For instance, *D. everestianus* has been previously reported only in northwestern China and Nepal at altitudes of 2600 m to 4700 m. In contrast, *I. acutitarsus* primarily inhabits mixed forest areas at altitudes of 1600 m to 2700 m, including regions in Southwest China, Chinese Taiwan, Nepal, Burma, Japan, and India [22]. In this study, Tanggu town, located in the northwest of Jiulong County, is a typical alpine pure pastoral area with an altitude ranging from 3050 m to 5424 m, which is suitable for the survival of *D. everestianus*. Xia’er town has an average altitude of 2700 m, with a mountain warm temperate and alpine sub-cold climate, which provides an ideal environment for the survival of *I. acutitarsus*.

For SFGR identification, according to Fournier’s criteria, the *ompA* gene is a specific protein gene for SFGR [23]. If the *ompA* gene is detected, it can be identified as SFG*R*. If not, SFGR must meet two of the following four criteria: (1) 16S rRNA homology > 98.80%; (2) gltA homology > 92.70%; (3) *ompB* homology > 85.80%; and (4) geneD homology > 82.20%. Thus, in this study, the *ompA* and *ompB* genes were used as target genes to detect SFGR infection in ticks from Jiulong County.

Additionally, in the study, it was revealed that *R. raoultii* accounted for a high proportion of SFG*R*. The SFGR infection rates in *I. ovatus*, *I. acutitarsus, R. microplus*, and *D. everestianus* ranged from 62.50% to 67.31%, with no significant differences, indicating that these four ticks are dominant SFGR-carrying species in Jiulong County. The *R. raoultii* infection rate in *D. everestianus* was significantly higher than previously reported by our laboratory (47.60%) [24]. Notably, the infection rate in semi-agricultural areas (70.60%) was significantly higher than in pure pastoral areas (45.10%) in Jiulong County, as reported in our previous findings in Shiqu County, Ganzi Prefecture, which may be related to differences in vegetation types between agricultural and pastoral areas [24].

Notably, in recent years, Japanese Spotted Fever (JSF) has emerged as the predominant tick-borne *Rickettsial* disease in China. JSF is a disease caused by *R. japonica*. In China, since its first discovery in Hainan Province in 1989, the epidemic range and number of infections have expanded significantly [25]. Currently, human cases of Japanese Spotted Fever have been reported in 14 provinces. Between 2021 and 2024, the number of reported cases nearly doubled [26]. In 2021, the first JSF case resulting in death—due to multiple organ failure and disseminated intravascular coagulation (DIC)—was reported in Zigui County, Hubei Province [27]. Rather, the regions where *R. japonica* was detected were mainly the humid mountainous areas of central and eastern China, and the infections exhibited a clear seasonal trend, with cases primarily occurring from April to November and peaking in autumn. Additionally, the study found that the high-risk population for JSF mainly consists of rural residents aged over 55. However, in this study, *R. japonica* was not detected in ticks. Collectively, given the high infection rates of *R. raoultii* in *I. ovatus*, *I. acutitarsus*, *R. microplus*, and *D. everestianus* in Jiulong County, it is crucial to investigate whether local residents are infected with *R. raoultii*.

In addition, there are still some limitations in this study: especially, the methods of collection and the host species are important factors that affect the results of tick species identification. The reasons are as follows: firstly, the developmental stages of one-host ticks, such as the tiny *Ixodes* ticks, occur entirely on a single host; secondly, over 90% of a tick’s life cycle is spent in a non-parasitic stage, with ticks typically inhabiting environments like grasslands and forests; thirdly, two-host and three-host ticks, such as *I. ovatus*, *I. acutitarsus*, and *D. everestianus*, drop off after feeding at each developmental stage and then seek a new host for the next stage. Future work should involve flagging methods or increase the variety of hosts by collecting ticks from the surfaces of sheep, horses, dogs, plateau pikas, plateau marmots, etc. Moreover, expanding the collection areas beyond both pure pastoral and semi-agricultural-pastoral areas in Jiulong County would likely yield a more comprehensive understanding of the types and distribution of ticks in the area.

## 5. Conclusions

In summary, this study represents the first systematic investigation of tick species infesting yaks and their infectious status with SFGR in Jiulong County, Sichuan Province, China. The results indicate that *I. acutitarsus*, *I. ovatus*, *R. microplus*, and *D. everestianus* are important vectors of *R. raoultii* in this area. The high prevalence of SFGR in ticks in Jiulong County greatly increases the risk of human infection. In the future, more research should focus on strengthening the surveillance of ticks and the epidemiological study of *R. raoultii*. This is essential for understanding the potential transmission risks and for developing effective prevention and control strategies to protect both human and animal health.

## Figures and Tables

**Table 2:** The infection rates of SFGR in ticks from different regions.

Location		Number of Positives	Number of Samples	Positive Rate (%)	SFGR Genotype Infection Rate (%)	
					*R. raoultii*	*R.* spp.
Pastoral area	Tanggu	69	153	45.10	30.07 (46/153)	15.03 (23/153)
	subtotal	69	153	45.10	30.07 (46/153)	15.03 (23/153)
Farming and Pastoral area	Sanyanlong	114	176	64.77	64.77 (114/176)	0.00 (0/176)
	Xia’er	191	256	74.61	48.44 (124/256)	26.17 (67/256)
	subtotal	305	432	70.60 **	55.09 (238/432)	15.51 (67/432)
Total		374	585	63.93	48.55 (284/585)	15.38 (90/585)

** Indicates that the difference between the same column is extremely significant (*p* < 0.01).

**Table 3:** Infection rates of SFGR in different tick species.

Tick Species	Distribution Location	Number of Positives	Number of Samples	Positive Rate (%)	95% Confidence Interval (%)
*R. microplus*	Xia’er, Sanyanlong	196	308	63.64	58.00–69.00
*I. ovatus*	Xia’er, Tanggu	123	193	63.73	56.50–70.50
*I. acutitarsus*	Xia’er	35	52	67.31	52.90–79.70
*D. everestianus*	Tanggu	20	32	62.50	43.70–78.90

## Data Availability

Available from the corresponding author on reasonable request, subject to compliance with institutional review board protocols.

## Supplementary Materials

The following supporting information can be downloaded at: https://www.mdpi.com/article/10.3390/ani15070975/s1.
