# Supplementary material for: Molecular Detection of SFGR in Ticks Collected from Yaks in Jiulong County of Sichuan Province, China
Source: Animals (Basel). 2025 Mar 28;15(7):975. doi: 10.3390/ani15070975 (PMC11988138; doi:10.3390/ani15070975)
Supplement: Supplementary file 1 [file animals-15-00975-s001.zip › animals-3495187-supplementary.pdf]

>Seq1 [organism= *I. ovatus* Jiulong1]
GGGGGATTTTTCCGGTGAGGTCGGATATATATCAGAGAGGAGAATTTTGTCG
ATTCTACCTCGTTTTGACTGTGTCGGGTCGTGGGCAGAACGCACGATAAAC
GTCTTTTCGGAGCAAATGGAATAACGAGGAAACGAAAAAAAAAACAACCT
GCACCCCGGGGCCTTTAAAAGGCGGGGGGTGGTTTCCTTATTTCCGAATTC
CCACGCCCATTTCCCTGACCGAAGGAACGAAAAAAATTACCCCATGGGAA
AAAAGGGAAAAAACAATGGTTCTCCTTTCCTTAAAGGCAAGGCCGGGGGG
GAAAAATACCCCCATTTTTTACCCCTTTCCAACCCTCCATTAACCGAAATTG
GCCAAGAAACCCCGTTTCCTTTTTTCTGAAGAATGGGGAACTTTCAGCTTT
CCTTTCCTCCCTGGTTTTTCAGGGCCGAAAGTGGGTTCCCAAACCAAGAAA
CCCAACCCCAGGAAACTGGGACCGATTTTCCTGTTTAAACCCCGCGCCCAA
GGGTTTGTTGTTTTTCCCGGGTCCTTTCCTCCCCCGAATAAAAAAACCCGT
GAATGCCTCGTCGCCCTCCCTCCATCCTAGAACCTCCCCGCCCCCATGAATA
CCCGCACTTACCCACTAGGGAATGCCGGTGGAAATGCGATGCTACTTTCTC
CTGTTGGACGAAATTTCTATCTCGGAGGGAAACGAGAAGCTGTGCATTTTG
AGAAAAGAACTGTTTTTCCTCGTGCCTGTGTGCGATGAGAGACCGTTTTTC
TCTACATATCATTGTCGACTCAGATCAGCGAAGCCACGGGAGACCGAGACC
GCATGTACGTTTA
>Seq2 [organism= *I. ovatus* Jiulong2]
CTACGGTCTCTGGTGAGTCGGATCATATATCAAGAGAGGAGAATTTTGTCGA
TTCTACCTCGTTTTGACTGTGTCGGGTCGTGGGCAGAACGCACGAAAAAC
GATAAACGTTTTTTCGGAGCAAATGGAATAACGAGGAAACGAAAAAAAAA
AAAACCTGCACCCGGGCGCATTGAAATGCCGGGGGGTGTTTGCGTAGTTTC
GGAATACCACTGCCGATTCTCGTGACGGAGGGGGCAAATGAAGTTCCCCAA
TGGGAGAAAAGGAAAAAAAAAGTGTCTTTCTTTCTCTGAAGGCGAGGGGG
GGGGGGAAAAAAACACCAATTCTTAACCCGTTTTGAGCCTTCGATGAGC
GGAGATTGACGAGGAAACTCGGTTTCTTTCTTTTTTTTTTTTGAGGATGGGG
GAAATTTCCCCCCCCTTTTCTCCCGGGTTTTTCGGGGGCGGAAAGGGAATT
CCAAAACAAGGAATGCAAACCCCAGGAAACTGGCCCGGATTTGCCCTTTT
AAACCCCCCCCCCAGGGTTTTGTTTGTTTTTCCCGGTCCCTTCCCCCCCCGG
AGGAAAAAACCCGTTTGAGTGCGCCCTACCCTTCCTCCATCCTAAAACCTC
CCCGTCCCCGACGAATACTGGCGCCCTCCTTTAGGGGAACGCCGTTGGAAA
TGCGATGCTCCTTTCTTCTTGTGGACGAAATTTCAATTCCCGGAGGGAAAC
AAGAAGCTGTGCATTTTGCGAAAAGACTGTTTTTTCTTCGTTGCTTGTGTTG
CGAAGAAAGAGAACGTTTTTTCCTCTTACAATATCATTGTCGACCCAAATC
AGGCGAGCCA
>Seq3 [organism= *I. ovatus* Jiulong3]
CGGGGCCTGGTGGGGATGGTCGGATCTATATCAGAGAGGAGAATTTTGTCG
ATTCTACCTCGTTTTGACTGTGTCGGGTCGTGGGCAGAACGCACGAAAAAC
GATAAACGTTTTTTCGGAGCAAATGGAATAACGAGGAAACGAAAAAAAAA
AAAAACACGGGCCCCGGGGGCCTTTGAAAGGCGGGGGGGGGTTTGCGTA
GTTTCGAAAAACCCCGGCCAATTCCCGGGAGGGGGGGAGCAAAGGAATTT
CCCCAAGGGGAAAAAAGGAAAAAAAAAGGGTTTTTCTTTCCTTAAAGGGG
AGGGGGGGGGGGAAAAAAACCCCATTTCTTACCCCGTTTTGACCCTTCA

AGGAGGGGAAATTGACAAGAAACCCCGGTTTCTTTTTTTTTTTTTCGGGGG
ATGGGGAAATTTTGCCCTCCCTTTTCCCCCGGGTTTTTCGGGGGGGGAAGG
GGATTTCCAAAACGGGGAAGGGGAACCCAAGGAAATTGGCCCGATTTTGC
GGGTTAAAACGGGCCCCCAAGGGTTTTGTTGGTTTTCCCCGGTCCCTCCCC
CCTCCGAAAAAAAAAACCTTTGGAGGGGGTCGAACCCTCCGCCCAGTCTA
GACCTTCCCGTCCCCAAGAATCTGGCGCCATCAGAGGGAACGCCGTGGAA
TGCAAGCTCTTTCTCTGGGACAATTTGATCCGGAGGAAACAGAGCTGTCAT
TGCAAAGACTGTTTCTCTGCTGGTGCATAAGAGACGTCTTCTCTAAAACCT
GCCACCAGACAGCAAG
>Seq4 [organism= *I. ovatus* Jiulong4]
GAGCGGTCTCTGTTGAGGTCGGATCATATATCAAGAGAGGAGAATTTTGTC
GATTCTACCTCGTTTTGACTGTGTCGGGTCGTGGGCAGAACGCACGAAAAA
CGATAAACGTTTTTTCGGAGCAAATGGAATAACGAGGAAACGAAAAAAAA
AAAACACATGCCCCCCGGCGCTCCCCTACGCCGGGGGTGGTTTGTTCATTT
TGGAAACACCACCGCTAAACTGACTGAAGGAGGGAACAAACGAAATCAC
CTCTTTTCATAAAAGGAAAAAAAAGAACCGTTTTAACTAACAAAGGGGA
GGGGCGGGGCGGAAAAAAAAACACATCCTTGCCCCTTTCCTCATGTCCTCA
TTCCTCGATCATTAATAGAAAACCAGCTCCTTTTTTTTTTTTTCCAAAGGATA
GCGCAAATTTCCCCCCTCCTCTTAACCAAGTTTTTAAGGCCGATTTTCCTCG
GTCTCACCGCGGATCGAAAGCTCAAGAAAAATGGGGAATTGTTTTATTCTT
AACCCCCGCCCTCAGGGGGTTCTTTTTTTTTACCCTCTCTTTTCCCCTCCCG
AGAAAAAAAAACCTTCTTTGGCGCCTCCCCCCCCCCCCATCCGTAAAACCT
TTCCCCCCCCCGAAAACACGGACCCCCCTTTTAGGGGCACCCGTTGGAAAT
TGATGCTCCTTTTTCTCGTGGACAAGATTTTAACTCGGAAGGAAAAAAAAA
ACTGTGTGCTTTTGAAAAAACACTGTTTTTCTTCTTTGCTTGTGTGGCGATA
TAAAAAAAGTTTTTTCCCTCACAATATCATTGTCCCCCTCCATAAGGCGCAA
ACCCCGAGACACGGCGGAGCCCCATTGTGATAGGGTTTAA
>Seq5[organism=*I. acutitarsus* Jiulong1]
CCCCCTTTCTTCTGTTTGAGTTCAGATCATATATCAAGAGAGGAGAGTCTTC
GGACTTCTGCCTCGTTTTGACTGTGTCGGGTCGTAGGCAGCACGCAATATC
TCGGCGTATCACTCACTCGCCTCGTCATCTTTTGGAGCGGAGCGACCTTCG
CTTGTTTTCTGGAATCACTGCCATGAAACGAAACCGTTGTTTTTTTGGGAA
ATCCGTCTTTCGTTTTTCTCATTCTCTTGGGGTTTCGCGGAAAACCTGACCG
CGCGGACGTGAGCCCAACCGATCCTGGAGGATTGTGGACGGTCGGTGACT
CCCCCCCCACCCTCAATGCGGAAAAAAAAGTGACAAAAAAAAAGTTCAG
GATTGAAAAAACGTGTTTCCTCTTGGCGTTGAAAGTGGGCCGGAAAAGTG
AAAAAGCAAAAAGGAACCGGCCCGTGGCTCGGGTTTTGCAAGTCTCCATG
GTTGGTGCTGGTGGCAAAAAAAAAAAATACTGAAATGCTTCTTACCCTTAC
CGTCAGTCTAAAACCTTCCCGTCCCCAATGAATACTGGACCCTTCCATTAGG
GAAAATGCCGTTGGATTTGTGGGTGCCCCCCCCCATCGGTAAAAAAACCCC
CCACCCCCCCTGTAAAAAAAAAACACATTCGGAACGAGCGCCCCAAAAAG
GAGAGGAGAACCGCGAGGAATACGGTTCTTGTCCTCTCGGCCCCTTTTCTG
GTGGTCGCGGTTTTTTTTTCGGGGGGGGGGGGCGTGACGTTGTATGGGAA
GGC

>Seq6[organism=*I. acutitarsus* Jiulong2]
CGCCGAACTTTCATCTTATCTCGGATCTATATCACCAGAGGAGAGTCTTCGG
ACTTCTGCCTCGTTTTGACTGTGTCGGGTCGTAGGCAGCACGCAATATCTC
GGCGTATCACTCACTCGCCTCGTCATCTTTTGGAGCGGAGCGACCTTCGCT
TGTTTTCTGGAATCACTGCCATGAAACGAAACCGTTGTTTTTGGGAAATC
CGTCTTTCGTTTTTCTCAGTCTCTTGGGGTTTCCCCGAAAATCTGAACGCGC
GGACCTGAACCCGACCGATCCTGGAGGATTGTGGACGGGCGGTGACTCAC
CCCCCATCCCCCATGCGGAAGAAAAACTGACGAAAAAAAAGTTCCCGATT
GAAAAAACGTGTTTCCTCCTGGCGTTGAAAGTGGGCCGGAAAAGTGGAGA
AGCGAAAACGAACCGGCCCCTGGCTCGGGTTTTGCGAATCCCCCTGGGTG
GTGCTGGAGGCGAGAAAAAGAAATAC
>Seq7[organism=*I. acutitarsus* Jiulong3]
CAGTCGTTAACTCATCGCTGTTGATTCGGATCTATATCAAGAGAGGAGAGTC
TTCGGACTTTTGCCTCGTTTTGACTGTGTCGGGTCGTAGGCAGCACGCAAT
ATCTCGGCGTATCACTCACTCGCCTCGTCATCTTTTGGAGCGGAGCGACCTT
CGCTTGTTTTCTGGAATCACTGCCATGAAACGAAACCGTTGTTTTTGGGA
AATCCGTCTTTCGTTTTTCTCAGTCTCTTGGGGTTTCGCGGAGAAGCTGAG
CGCGCGGACGTGAGCCCGACCGATCCTGGAGGATTGTGGACGGTCGGTGA
CTCACGCGCCAGCCTCGATGCGGAAGAAAAAGTGACGAGAAGAAAGTTC
AGGATTGAAGAAACGTGTTTCCTCTTGGCGTTGAAAGTGGGCCGGAAAAG
TGGAGAAGCGAGAAGGAACCGGCGCGTGGCTCGGGTTTTGCGAGTCTCCA
TGGTTGGTGCTGGTGGCGAGAGAGAGAGATACTGAGATGCGTCGTAGCCAT
ACCGTCAGTCTAAGACCTTCGCGTCCCCGATGAATACTGGACCCATCCAGT
AGGGGAAATGCCGTTGGATTTGTGGGTGCCCCCCCAGATCGGTAAACGAA
CGCCCCACCCCGCCTGTAAAAAAAAAAACGCATTCGGAACGAGCGAGCCA
AGAAGGAAGGAGGAACCGCGAGGAATACGGTTCTTGTCTTCTCGGCTCCG
TTTCTGGTGGTTGCGGTTTTTGTTCGGTGGGGTGGGTGCGTGACGTTGTAT
GGGAAGGGCCGTTCACAAAAGTGGAAATCCGATCTCTCCGGCGGCGCGCA
CACCTTCTTTGAGTTGGGTGAAGGAGCGCGACGCGGGGAAAGAGAGAGA
GACGACCTAAAAAAAAAACATTCTGTCTGACATCGATAAGGCAAATGTAGT
TGACTT
>Seq8[organism=*R. microplus* Jiulong 1]
CTTTTTTTCGGTTTGAGGGTCGGATCCATATCAAGAGAGCCTTCGGCGCAC
AAGGGAACGTGAGCCGTCGACTCGTTTTGACCGCGTCGGCAACACGGACA
GCACGCTGAACACCTCACAGCGAGCGCCAACAGCGGCCACTCAAGGGCG
AGACGGTGGCGACCGTCGTGCCAGAGCCCAACCGAAACGGGGGCGACCG
ACTGCATTGAGGATGTGGCACCTCGTTGAGACCGCCGCAGGACTTCGAGT
CGGAAGGAAGCCTGCAGGGAAAGTGCGGTCGAGGTTGCGTACTCCTCTCT
GCGACCGGGCGCGCAAGAGCTGCGAGAGCCACGGACGCGCAACTTTAAC
GCACGGTAAACACGAGGAGCGAAAGCCGGCCAGCAAAGCTTCTCCAGCC
GTGCGCAAAGTGCGCGAGATCGCAGCCTTGCGTTGCGCTTGTTGCCCTCGA
AGTAAGCTGGGTGTCCCGTAGACCGGGCGCTCGAACACGCTGCGGGGCCG
TGCCTCCTCCAGGCTTTGCCGCGCGAACAGGGAACGTTCGCGCGCAAAGC
GCAGGGAGGTGAGGAGGCTGCGCCCGACGTTTGCGGTTCGCTGCGTACGC

GGTTGATGCGGAGAGCACGGCGCGACGACTTGCCGCGAAGCGGAAAAAG
TCTCCCGCACGAGTTGGCGAAACGTTGGCGAAGCTTAAGGCGTTCTCGTC
GTAGTCCGCCGTCGGTCTAAGTGCTTCGCAGTTCCCGTCCCGTTCAAAAAA
CTGGGCCACTCCAGTTGGGGCGGGGGCGACGCTACACGAGACGATGCCTC
TCGCCAGGCTGCGTGGCTGCCCTTGCGGCGGCGGCGACTGGCCTCGGCGG
TGTTTGGGCTTTCGACACGGTCGTTTATCACGCAACTGCTCGGACGACGCA
CGCGCGCAGCGGAATGCCGCTTGCCAGCCTTGTGAAGATGTGACCCTGTAC
AGGGTTGCGGGCGCACTTGGTAGGGCGTCGTACTCGGTTCGCGATGGTTTA
CGAACGTGTCCCGTCACTTCACGTCACACCGGGTTGTGCGCCGCACGCGT
GCAGCGGGGAAGCTGATTGTCAATCTTGTGAGAAAGGCCCTGAACCAGGT
TGC
>Seq9[organism=*D. everestianus* Jiulong 1]
CACGTTTTCTGTTGACGTCGGATCACATATCAAGAGAGCCTTCGGCGCACA
GGGAACGTGCGTCCGTCGACTCGTTTTGACCGCGTCGGCATCACGGACAGT
ACGTTGAGTGCTGAAGCCACGCGCCAGCGGCCTCACGTGAGAGAGACTGT
GGCGAACTAAACTGTTGTGCCAAAACTTCGCAGAGACGGAAACGAGGCAT
TGTACTACTGCAGCGCGACGAGCGCGCGCCTCCGAAGAGACCGCCGCAGG
GTGGAGTCGGACACCTGCAGGGAAAGCGCGGTCCGAGCGCGAGGCGCGA
ACGTCTGTTGCAACAGCAGTGGCGCGCACGTTTGCGAGAGAGTCGGAAGC
GCATGCTTGCATGCACGGTTAACGCGGGAAGCACACTTGTTGGTTCCTGCG
CCGTGAGCGAAGCGAGCGCGATCGCGCGTTGCGTTGTTTGCCTTCGGAGTA
CGTCGAGCTCTAGCAAAAGGTCGCTCGAGCGTCCGCGTCACCGCACGGGT
GCAGGCGCCCTGGTCCAAGTTTCGTCGCAAGAGTAGGAATCGGAAAAGAT
TCTTGCGCGGAGCAGAAAGGACAAGGGTGCGCCCCAAAGCGGTAGTGATG
CGTTTACGCGAGTGTGCCGTCTGCGAGCGCGAAGAAAACGGTACGACGGA
TTAGTCGCCGCGAAGCGGAAAAATGTCTTTCTCGAAAGCGTGAGTTTGCCC
GCTGGCGGAGCTGAAGCGTTCCGTCGTAGTCCGCCGTCGGTCCAAGTGCTT
CGCAGTCTCTGCCCCGAAAAGACTGGGTCCTTCCAGTTGGGGTAGGGGCG
ACGCTACACGAGATGATGCCTCCTGCCAGGCTAGAGCCGTCCTGCGGCAGC
CGCTGAAGCGGCGCGCTGCGAGGGTGGCACGCCTCGGCGGTGTTTGGGCT
TCAGACATGGTCGTTTGCCACGCAACTGCTCGTGCGCCGCACGCGCGTGG
GCGGTTTTTAAACCGCCTGCCAGCCTCGTCTATAAGTAGCTCCGTGTTGGG
CGAAGGACGTGGTAGGCGTCGTACTCGGTTTGCGCTGGGCTTACGAACGT
GTCGAGTCCTTTCCAGCTCGCTGGAATGGGCGAAGCGATGTATCGGTTGTC
GACCTCAGATCAAGGCGGAGCCAAGGAAGACCCAGGGCCCGCAATGAAA
